# Assessment of CVD Risk Factors in Secondary Prevention after Ischemic Stroke Using the ICF

**DOI:** 10.3390/ijerph19063368

**Published:** 2022-03-12

**Authors:** Mateusz Lucki, Ewa Chlebuś, Agnieszka Wareńczak, Przemysław Daroszewski, Przemysław Lisiński

**Affiliations:** 1 Department of Rehabilitation and Physiotherapy, University of Medical Sciences, 60-545 Poznań, Poland; ewachlebus@ump.edu.pl (E.C.); agnieszka.warenczak@gmail.com (A.W.); plisinski@vp.pl (P.L.); 2 Department of Organization and Management in Healthcare, University of Medical Sciences, 60-545 Poznań, Poland; dyrektor@orsk.pl

**Keywords:** CVD, ICF, ischemic stroke, risk factors, secondary prevention

## Abstract

Background: Patients after undergoing ischemic stroke have a high risk of further cardiovascular disease (CVD) incidents. Monitoring risk factors is critical to prevent the recurrence of CVD. Objective: The aim of the study was to determine differences in the incidence of risk factors for CVD in a post-ischemic stroke patient group (SG) compared to the control group, which had not undergone ischemic stroke (CG), and to characterize them using the ICF (International Classification of Functioning, Disability and Health) classification system. Materials and Methods: The incidence of risk factors for recurrent CVD events were retrospectively analyzed in 55 patients in SG and 55 patients in CG. The results were translated into categories from the ICF classification system. Results: Atrial fibrillation (*p* = 0.013), carotid artery stenosis > 50% (*p* < 0.001), LDL > 71 mg/dL (*p* < 0.001), heart rate > 80/min (*p* = 0.007), taking NOAC (*p* = 0.008) and NSAIDs (*p* < 0.001) as well as nicotinism (*p* = 0.001) were significantly more common in SG compared to CG. The value of the distribution of the total incidence of CVD risk factors were observed to be higher for SG than for CG. In SG, both for males (*p* < 0.001) and females (*p* < 0.001) more risk factors for recurrent CVD incidents were observed compared to CG. Conclusions: Patients in SG differ in the occurrence of risk factors for CVD event compared to CG. The use of a single tool, such as the ICF assessment sheet, can be useful in assessing and analyzing risk factors for recurrent CVD events. This may help to reduce the risk of subsequent CVD events in secondary prevention.

## 1. Introduction

Patients who undergone ischemic stroke are at a high risk of further cardiovascular disease (CVD) events [1]. The overall 1-year and 10-year risks of recurrence were 4% and 13% following first-time ischemic stroke. Furthermore, the 1-year and 10-year risks of mortality were 17% after a first-time ischemic stroke and 25% and after a recurrent stroke. In addition, 15% of patients may have an additional heart attack, and a similar percentage of patients will die from the vascular disease [2]. According to World Health Organization (WHO) experts, nearly 80% of repeated CVD events could be avoided if the main risk factors were removed [3]. Strong evidence suggests that effective prevention strategies may reduce the risk of recurrent cardiovascular events, thereby decreasing mortality rates and improving morbidity, disability outcomes, and quality of life—provided that intervention is administered early enough [4]. The commonly known modifiable risk factors for recurrent CVD are arterial hypertension, atrial fibrillation, diabetes, dyslipidemia, abnormal body mass index (BMI), carotid artery disease, depression, insomnia, smoking, and alcohol abuse [5,6,7,8,9,10,11,12,13,14,15,16,17,18,19,20]. It is worth adding that ischemic stroke may be caused by hematological disorders or resistance to acetylsalicylic acid [21]. Patients with multiple risk factors have an increased risk of a recurrent CVD event [22]. For this reason, it is necessary to monitor these factors at the same time.

The International Classification of Functioning, Disability, and Health (ICF) is an excellent instrument that transforms into simplified, categorized charts information from widely used scoring systems to assess cardiovascular events’ risk. The ICF classification is an international tool that comprehensively assesses health disorders [23]. Recoding data into ICF classification categories enables their presentation in a universal, legible graphic form, which facilitates making the right clinical decisions [24]. To date, few articles on applying the ICF classification in secondary CVD prevention have been published [25].

## 2. Objectives

The purpose of these examinations was:

(1) Assessment of the frequency of risk factors for recurrent CVD events between the group of patients after ischemic stroke (SG) and the control group without previous CVD (CG) events.
(2) Use of ICF classification categories to represent the frequency of risk factors for recurrent CVD events between SG and CG.

## 3. Materials and Method

### 3.1. Study Design

The study was retrospective and consisted of two stages. The first stage was to analyze the incidence of risk factors for a recurrent CVD incident based on the clinical examination, and results of the additional tests included in medical documentation such as laboratory tests, medical imagings, measurements of blood pressure, and electrocardiograms. The second stage was to recode the obtained results into ICF classification categories and present them in a graphical form.

The study groups consisted of the group after ischemic stroke (SG) and the control group (CG) without stroke. The study groups were analyzed in the same way.

The SG consisted of 55 patients (mean age, 63.2 ± 8.8) hospitalized in the Department of Neurological Rehabilitation in the Wiktor Dega Memorial Orthopedic & Rehabilitation Teaching Hospital in Poznań within 14 days of stroke between September 2020 and January 2021.

The study inclusion criteria were as follows: (1) ischemic stroke confirmed by medical imaging (2) patients hospitalized within 14 days of the stroke in the neurological rehabilitation department, (3) a completely reliable medical record which included accurate information about analyzed risk factors (clinical examination, results of the additional tests such as laboratory tests, medical imagings, measurements of blood pressure, and electrocardiograms) kept by the medical staff at the hospital, and (4) a declaration of the regular use of the pharmacotherapy of chronic diseases under the supervision of a medical doctor by patients. If patients did not meet all of the above inclusion criteria, they were excluded from the study.

The control group consisted of 55 volunteers similar in age to the study group (mean age 65 years, 9 ± 6.3) recruited from among hospital patients who: (1) had no history of stroke and (2) had a completely reliable medical record which included accurate information about analyzed risk factors (clinical examination, results of the additional tests such as laboratory tests, medical imagings, measurements of blood pressure, and electrocardiograms), (3) declared the use of regular pharmacotherapy of chronic diseases under the supervision of a medical doctor. If volunteers did not meet all of the above inclusion criteria, they were excluded from the study. Recruitment for the control group was carried out on the hospital’s website.

### 3.2. ICF Profile

The assessed risk factors of CVD were recoded into appropriate categories and qualifiers in accordance with the ICF classification.

The effect of depressive disorders on the risk of a recurrent CVD event was assessed using ICF category b152: emotional functions. The following Beck Depression Inventory (BDI) scores were used to measure the severity of depression [26]: qualifier 0: BDI total score 0 to 11—no depression; qualifier 2: BDI total score 12 to 19—mild depression; qualifier 3: BDI total score 20 to 25—moderate depression; qualifier 4: BDI total score 26 to 63—severe depression.

The effect of sleep disturbance on the risk of a recurrent CVD event was assessed using ICF category b134: sleep functions. The following criteria were used to measure the severity of insomnia [6]: qualifier 0—no sleep disturbance (sleep time 6–9 h); qualifier 4—sleep disturbance (sleep time < 6 or >9 h).

The increased risk of CVD related to heart rate (HR) was estimated using ICF category b4100: heart rate. The following criteria were used to quantify heart rate disorders [7]: qualifier 0—HR < 80/min; qualifier 4—HR > 80/min. Heart rhythm disorders were encoded as ICF category b4101: heart rhythm. The following criteria were used [8]: qualifier 0—normal sinus rhythm; qualifier 4—atrial fibrillation.

The effect of carotid artery stenosis on the risk of a recurrent CVD event was assessed using ICF category b4150: functions of arteries. The following criteria were used [9]: qualifier 0—<50% carotid stenosis; qualifier 3—50% to 69% carotid stenosis; qualifier 4—>70% carotid stenosis.

The effect of increased blood pressure (BP) on the risk of a recurrent CVD event was assessed using ICF category b4200: increased blood pressure. The following BP values were used [27]: qualifier 0—BP < 130/80 mm/Hg; qualifier 1—BP > 130/80 mm/Hg; qualifier 2—BP > 140/90 mm/Hg; qualifier 3—BP > 160/90 mm/Hg; qualifier 4—BP > 180/110 mm/Hg.

The effect of liver and renal impairment on the risk of a recurrent CVD event was assessed using ICF category b4301: metabolite-carrying functions of the blood. The following criteria were used to classify renal impairment [28]: qualifier 0—estimated glomerular filtration (eGFR) > 90 mL/min/1.73 m^2^; qualifier 1—eGFR 60–89 mL/min/1.73 m^2^; qualifier 2—eGFR 30–59 mL/min/1.73 m^2^; qualifier 3—eGFR 15–29 mL/min/1.73 m^2^; qualifier 4—eGFR < 15 mL/min/1.73 m^2^, and liver impairment [12]: qualifier 0—bilirubin level < 2× the upper limit of normal (ULN) and ALT(alanine transaminase)/AST(aspartate transaminaze)/ALP(alkaline phosphatase) < 3× ULN; qualifier 4—bilirubin level > 2× ULN and ALT/AST/ALP > 3× ULN.

Patients receiving anticoagulants have got an increased risk of bleeding [13]. This parameter was encoded as ICF category b4302: functions related to the coagulation of blood. If taking VKA (vitamin K antagonist), the following values were used: qualifier 0—NO; qualifier 4—YES. If taking NOAC (non-vitamin K antagonist), the following values were used: qualifier 0—NO; qualifier 4—YES.

The effect of impaired glycemic control on the risk of a recurrent CVD event was assessed using ICF category b5401, carbohydrate metabolism. The following HbA1c(glycated hemoglobin 1c) values were used [29]: qualifier 0—HbA1c < 7%; qualifier 4—HbA1c > 7%.

The effect of LDL-C (low-density lipoprotein cholesterol) levels on the risk of a recurrent CVD event was assessed using ICF category b7302, lipid metabolism. The following LDL-C values were used [30]: qualifier 0—LDL-C < 55 mg/dL; qualifier 2—LDL-C 55 mg/dL–70 mg/dL, qualifier 3—LDL-C 71 mg/dL–115 mg/dL, qualifier 4—LDL-C > 116 mg/dL.

Alcohol consumption is an additional risk factor associated with an increased risk of a recurrent CVD event. This risk factor was assessed using ICF category e1100, food: alcohol consumption. The following criteria were used [31]: qualifier 0—alcohol intake per day < 10 g; qualifier 4—alcohol intake per day > 10 g.

The increased risk of CVD related to NSAID (nonsteroidal anti-inflammatory drugs) [17] and to smoking [18] was estimated using ICF categories e1101, drugs and e1109, products or substances for personal consumption, respectively. The following criteria were used: qualifier 0—NO; qualifier 4—YES.

The ICF qualifiers of the assessed CVD risk factor category are presented in percent distribution using graphical color coding to highlight differences in risk factor frequency between SG and CG. The following qualifier designations were adopted: qualifier 0—no risk factor is present if the percentage distribution ranges from 0% to 4%, color code: dark green; qualifier 1—low prevalence of the risk factor if the percentage distribution ranges from 5% to 24%, color code: light green; qualifier 2—moderate prevalence of the risk factor if the percentage distribution ranges from 25% to 49%, color code: yellow; qualifier 3—high prevalence of the risk factor if the percentage distribution ranges from 50% to 95%, color code: orange; qualifier 4—extremely high prevalence of the risk factor if the percentage distribution ranges from 96% to 100%, color code: red.

### 3.3. Statistical Analysis

The data analysis was carried out using Statistica v. 13.1. The parameters of descriptive statistics are reported as mean values with standard deviations (SD) and median, minimum, and maximum levels. The categorical variables are presented as counts and frequencies. The Shapiro–Wilk test was used to assess the normality of the distribution of test scores. The significance of differences between results or both groups was evaluated based on the non-parametric Mann–Whitney test. The chi-squared test was used to compare differences between groups in terms of categorical variables. *p*-values less than 0.05 were considered to be statistically significant. The data such as increased blood pressure and atrial fibrillation from the first twenty subjects from both groups were used to determine the required sample size with a power of 80% and a significance level of 0.05 (two-tailed). The sample size software estimated that 43 patients were needed.

### 3.4. Ethical Approval

The study was approved by the medical ethics committee of the Poznan University of Medical Sciences (Approval No. 174/21 of 11 March 2021) and was performed in accordance with the Declaration of Helsinki. Written informed consent was obtained from study participants after an explanation of the aim and methodology of the study. The study was registered in the Clinical Trial Registry: NCT04590287 https://clinicaltrials.gov/ct2/show/NCT04590287 (accessed on 19 October 2020).

## 4. Results

### 4.1. Study Population

The study groups were not different in terms of age and sex distribution. In the SG, significantly more common was arterial hypertension (*p* < 0.001), coronary heart disease (*p* = 0.017), chronic heart failure (*p* < 0.001), and carotid artery disease (*p* < 0.001) than compared to the CG. Detailed characteristics of the study groups are shown in Table 1.

### 4.2. Risk Factors for Recurrent CVD Incidents

Table 2 presents an analysis of the frequency of CVD risk factors in secondary prevention depending on the history of ischemic stroke and gender.

#### 4.2.1. SG vs. CG

In SG heart rate value > 80/min (*p* = 0.007), atrial fibrillation (*p* = 0.013), carotid artery stenosis ranging from 50% to 69% (*p* < 0.001) and >70% (*p* < 0.001), elevated arterial blood pressure > 140/90 mmHg, LDL values ranging from 71 mg/dL to 115 mg/dL (*p* < 0.001) and >116 mg/dL (*p* < 0.001) as well as more frequent use of NOAC (*p* = 0.008), NSAIDs (*p* < 0.001) and smoking (*p* = 0.001) were significantly more commonly observed compared to CG.

#### 4.2.2. SG vs. CG in Relation to Female Gender

Regarding the female gender in SG carotid artery stenosis ranging from 50% to 69% (*p* = 0.011) and >70% (*p* = 0.005), elevated arterial blood pressure > 140/90 mmHg (*p* = 0.004), LDL values ranging from 71 mg/dL to 115 mg/dL (*p* < 0.001) and >116 mg/dL (*p* < 0.001), as well as smoking (*p* = 0.036) were significantly more commonly observed compared to CG.

#### 4.2.3. SG vs. CG in Relation to Male Gender

Regarding the male gender in SG heart rate value > 80/min (*p* = 0.007), atrial fibrillation (*p* = 0.005), carotid artery stenosis ranging from 50% to 69% (*p* = 0.005) and >70% (*p* = 0.002), elevated arterial blood pressure > 140/90 mmHg (*p* = 0.004), LDL values ranging > 116 mg/dL (*p* < 0.001) as well as use of NOAC (*p* = 0.038), NSAIDs (*p* = 0.004) and smoking (*p* = 0.013) were significantly more commonly observed compared to CG.

### 4.3. CVD Risk Factor Profile in Secondary Prevention According to the ICF Classification

The ICF classification categories presented in Table 3 indicate risk factors that need to be monitored in secondary prevention of CVD. The chart represents the percentage distribution of these risk factors according to the SG and CG results.

#### 4.3.1. SG vs. CG

For the category “extremely high prevalence”, risk factors such as depression, sleep disturbance, heart rate and heart rhythm disorders, carotid artery stenosis ranging > 70%, elevated arterial blood pressure > 180/110 mmHg, glomerular filtration rates < 15 mL/min/1.73 m^2^, abnormal liver function, NOAC, VKA and NSAIDs use, abnormal glycated hemoglobin levels > 7%, LDL values ranging > 116 mg/dL, as well as smoking were more frequently observed in SG compared to CG. The frequency of alcohol abuse was similar in both groups.

For the category “high prevalence”, risk factors such as carotid artery stenosis ranging from 50% to 69%, elevated arterial blood pressure > 160/90 mmHg, glomerular filtration rate ranging from 15 to 29 mL/min/1.73 m^2^, and LDL values ranging from 71 to 115 mg% were more frequently observed in SG compared to CG.

For the category “moderate prevalence”, risk factors such as elevated blood pressure, >140/90 mmHg were more frequently observed in SG, while LDL values ranging from 55 to 70 mg% and glomerular filtration rate ranging from 30 to 59 mL/min/1.73 m^2^ were more commonly observed in CG.

#### 4.3.2. SG vs. CG in Relation to Female Gender

For the category “extremely high prevalence”, risk factors such as depression, heart rate and heart rhythm disorders, carotid artery stenosis ranging > 70%, elevated arterial blood pressure > 180/110 mmHg, abnormal liver function, NOAC and NSAIDs use, abnormal glycated hemoglobin levels > 7%, LDL values ranging > 116 mg/dL, sleep disturbance, glomerular filtration rates < 15 mL/min/1.73 m^2^, as well as smoking were more frequently observed in SG compared to CG. The incidence of VKA use was higher in CG compared to SG.

For the category “high prevalence“, risk factors such as carotid artery stenosis ranging from 50% to 69%, elevated arterial blood pressure > 160/90 mmHg, glomerular filtration rate ranging from 15 to 29 mL/min/1.73 m^2^ and LDL values ranging from 71 to 115 mg% were more frequently observed in SG compared to CG.

For the category “moderate prevalence“, elevated arterial blood pressure > 140/90 mmHg was higher in SG, while LDL values ranging from 71 to 115 mg% and glomerular filtration rate ranging from 30 to 59 mL/min/1.73 m^2^ were more frequently observed in CG.

#### 4.3.3. SG vs. CG in Relation to Male Gender

For the category “extremely high prevalence”, risk factors such as depression, heart rate and heart rhythm disorders, carotid artery stenosis ranging > 70%, elevated arterial blood pressure > 180/110 mmHg, glomerular filtration rates < 15 mL/min/1.73 m^2^, abnormal liver function, NOAC, VKA and NSAIDs use, abnormal glycated hemoglobin levels > 7%, LDL values ranging > 116 mg/dL, as well as smoking were more frequently observed in SG compared to CG. The frequency of alcohol abuse was similar in both groups.

For the category “high prevalence”, risk factors such as carotid artery stenosis ranging from 50% to 69%, elevated arterial blood pressure > 160/90 mmHg, glomerular filtration rate ranging from 15 to 29 mL/min/1.73 m^2^, and LDL values ranging from 71 to 115 mg% were more frequently observed in SG compared to CG.

For the category “moderate prevalence”, elevated arterial blood pressure > 140/90 mmHg was higher in SG, while glomerular filtration rate ranged from 30 to 59 mL/min/1.73 m^2^ in CG. The frequency of LDL values from 71 to 115 mg% were similar in both groups.

### 4.4. Concomitance Occurrence of CVD Risk Factors

Table 4 shows a comparison of the total incidence of CVD risk factors. In SG compared to CG (*p* < 0.001), significantly more risk factors for CVD were observed, also in relation to gender, both in females (*p* < 0.001) and males (*p* < 0.001).

**Figure 1:**
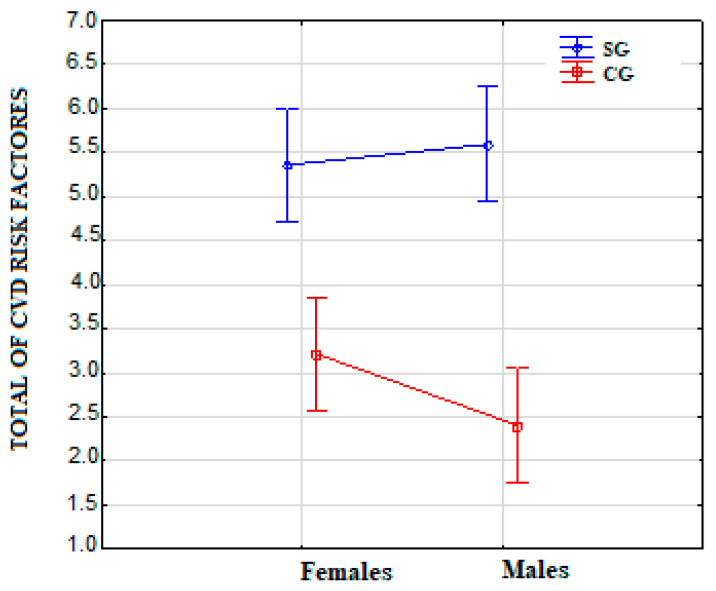
Distribution of the total of CVD risk factors. Note: CVD—cardiovascular disease; CG—control group; SG—post-ischemic stroke group.

The values of the distribution of the total risk factors for CVD were observed to be higher in SG than CG in relation to gender, both in females and males. The most significant values of the distribution of risk factors for CVD in SG were observed for males (Figure 1).

## 5. Discussion

Sequelae of cardiovascular disease are a major cause of death around the world and more than half of the patients with a history of stroke are at an increased risk of recurrent CVD incidents, including recurrent stroke, in particular [32] Moreover, according to Blanco-Rojas et al. [33], recurrent ischemic strokes of the brain are associated with a reduction in cognitive functions of the vascular type. Therefore, it is crucial to identify and monitor risk factors for recurrent CVD events in secondary prevention. In our study, the incidence of hypertension, coronary heart disease, chronic heart failure, and carotid artery disease was higher in patients with a history of ischemic stroke (Table 1). A study by Zhang et al. [34] yielded similar results. Risk factors are associated with the risk of recurrent CVD events and increased mortality and disability rates [33]. The incidence of risk factors for CVD in secondary prevention depending on the history of non-ischemic stroke and gender is provided in Table 2. The results show differences in risk factors between patients in SG and CG. These data suggest that atrial fibrillation and abnormal heart rate were more common in patients with a history of ischemic stroke and in relation to the male gender. It is consistent with the results obtained by Lip et al. [8], who determined that atrial fibrillation was associated with a high risk of recurrent CVD incidents. Wang et al. [35] demonstrated that patients with HR > 80/min have also a significantly higher risk of recurrent ischemic stroke. As shown in our research, carotid artery stenosis is also more common in patients with a prior history of ischemic stroke. This is in line with the results obtained by Orrapin et al. [9]. Blood pressure > 140/90 mmHg is another risk factor more commonly observed in SG compared to CG. As argued by Lewington et al. [36], abnormal blood pressure is associated with adverse events such as ischemic and hemorrhagic stroke, myocardial infarction, or sudden cardiac death. In our study, the use of NOAC and NSAIDs was significantly higher with a history of ischemic stroke and in relation to the male gender compared to CG. Our findings are in line with Gerner et al. [13] and Narum et al. [17], whose proved that the use of NOAC and NSAIDs is associated with an increased risk of intracerebral hemorrhage. In SG, we have observed significantly higher LDL values. Abnormal LDL values are strongly associated with increased risk of CVD incidents [15]. Active smoking may lead to recurrent CVD events. According to Epstein et al. [18], cessation of cigarette smoking after an ischemic incident is associated with decreased 5-year risk of stroke, myocardial infarction, or death. Compared to patients with CG, significantly more coexisting risk factors for CVD were observed in SG, both in females and males. The most significant values of the distribution of risk factors for CVD in SG were observed in males. According to Adams et al. [22], the more risk factors are identified, the greater the likelihood of a recurrent CVD incident.

When using the ICF classification in secondary prevention of CVD incidents, specific attention should be paid to the risk factors associated with extremely high incidence (qualifier 4), marked in red (Table 2). In relation to the female gender, particular attention should be paid to depression, heart rate, and heart rhythm disorders, carotid artery stenosis ranging > 70%, elevated arterial blood pressure > 180/110 mmHg, abnormal liver function, NOAC and NSAIDs use, abnormal glycated hemoglobin levels > 7%, LDL values ranging at >116 mg/dL, sleep disturbance, glomerular filtration rates < 15 mL/min/1.73 m^2^, and smoking. In relation to the male gender, particular attention should be paid to depression, heart rate, and heart rhythm disorders, carotid artery stenosis ranging at >70%, elevated arterial blood pressure > 180/110 mmHg, glomerular filtration rates < 15 mL/min/1.73 m^2^, abnormal liver function, NOAC, VKA and NSAIDs use, abnormal glycated hemoglobin levels > 7%, LDL values ranging >116 mg/dL, as well as smoking.

Percentage distribution of risk factors for relapse CVD events by ICF Category, presented in Table 3, provides information on the risk factors of a CVD event depending on the history of ischemic stroke. The ICF classification sheet enables the compilation of risk factors in one place by creating “dynamic charts” allowing for their comprehensive analysis and thus, facilitating the best decisions regarding secondary cardiovascular disease prevention.

## 6. Limitations

The limitations of our study were the retrospective character of the study. Another limitation of the study is that the sample size is relatively small for both groups. Another issue is the failure to consider BMI as a well known risk factor for recurrent CVD events due to the lack of data enabling its calculation based on the available medical documentation. Furthermore, some categories of ICF classification could only be represented as extreme qualifiers (4 or 0) without the possibility of using intermediate values.

## 7. Conclusions

Patients in the post-ischemic stroke group differ in the type and number of risk factors for a reoccurrence of a CVD event compared to gender and control group. The greatest distribution values of risk factors for CVD were observed in the male gender in the post-ischemic stroke group. As expected, patients in the post-ischemic stroke group have more risk factors for CVD incidents compared to the control group. The ICF assessment sheet collects commonly recognized CVD risk factors in one sheet, indicating which risk factors require special monitoring in clinical practice, which may simplify making clinical decisions. Using a single tool, such as the ICF assessment sheet, to monitor multiple risk factors for CVD may increase the effectiveness of preventative measures and thus, decrease the recurrence rate of cardiovascular events.

## Figures and Tables

**Table 1:** Characteristics of the study groups.

		SG	CG	*p*
Sex *n* (%)	Males	28 (50.9%)	28 (50.9%)	1.0 ^a^
	Females	27 (49.1%)	27 (49.1%)	
Age (years)	Mean ± SD	63.2 ± 8.8	65.9 ± 6.3	0.066 ^b^
	Median	63.4	67	
	Min–Max	42.7–78.9	47.0–77.0	
BMI	Mean ± SD	28.9 ± 4.6	28.6 ± 4.9	0.630 ^b^
	Median	28.7	28	
	Min–Max	18.6–37.8	21.0–42.8	
Comorbidites				
Hypertension	*n* (%)	50 (90.9%)	32 (58.2%)	<0.001 ^a^
Coronary heart disease	*n* (%)	16 (29.1%)	6 (10.9%)	0.017 ^a^
Chronic heart failure	*n* (%)	18 (32.7%)	7 (12.7%)	<0.001 ^a^
Diabetes melitus type 2	*n* (%)	24 (43.6)	17 (30.9%)	0.168 ^a^
Carotid artery disease	*n* (%)	30 (54.5%)	3 (5.5)	<0.001 ^a^

^a^ chi^2^ test; ^b^ Mann-Whitney test; BMI—Body mass index CG—control group; *n*—size of the sample; SG—Stroke group; SD—standard devation.

**Table 2:** Differences in the incidence of CVD risk factors depend on the history of non-ischemic stroke and gender.

		^SG^ vs. ^CG^			^Females^			^Males^		
		SG	CG	*p*	SG	CG	*p*	SG	CG	*p*
b152 Emotional functions—Depression [26]	*n* (%)	12 (21.8%)	5 (9.3%)	0.071	7 (25.0%)	4 (14.3%)	0.314	5 (18.5%)	1 (3.8%)	0.092
b134 Sleep functions—Insomia [6]	*n* (%)	12 (21.8%)	20 (36.4%)	0.092	7 (25.0%)	9 (32.1%)	0.556	5 (18.5%)	11 (40.7%)	0.074
b4100 Heart rate [7]	HR > 80/min, *n* (%)	25 (46.3%)	12 (21.8%)	0.007	15 (53.6%)	10 (35.7%)	0.179	10 (38.5%)	2 (7.4%)	0.007
b4101 Heart rhythm [8]	Atrial fibrillation, *n* (%)	15 (27.3%)	5 (9.1%)	0.013	6 (21.4%)	4 (14.3%)	0.485	9 (33.3%)	1 (3.8%)	0.005
b4150 Functions of arteries [9]	Stenosis 50–69%, *n* (%)	15 (27.3%)	1 (1.8%)	<0.001	8 (28.6%)	1 (3.6%)	0.011	7 (25.9%)	0 (0.0%)	0.005
	Stenosis > 70%, *n* (%)	15 (27.3%)	0 (0.0%)	<0.001	7 (25.0%)	0 (0.0%)	0.005	8 (29.6%)	0 (0.0%)	0.002
b4200 Increased blood pressure [27]	>140/90 mmHg, *n* (%)	50 (90.9%)	32 (58.2%)	<0.001	25 (92.6%)	16 (59.3%)	0.004	25 (92.6%)	16 (59.3%)	0.004
b4302 Metabolite-carrying functions of the blood [11,12]	eGFR (mL/min/1.73 m^2^) < 15, *n* (%)	6 (10.9%)	5 (9.1%)	0.753	3 (10.7%)	4 (14.3%)	0.686	3 (11.1%)	1 (3.8%)	0.299
	Bilirubin > 2x ULN, ALT/AST/ALP > 3x ULN, *n* (%)	5 (9.1%)	1 (1.8%)	0.092	4 (14.3%)	1 (3.6%)	0.16	1 (3.8%)	0 (0.0%)	0.313
b4303 Functions related to the coagulation of blood [13]	VKA, *n* (%)	11 (20.0%)	9 (16.4%)	0.625	6 (21.4%)	7 (25.0%)	0.752	5 (18.5%)	2 (7.4%)	0.224
	NOAC, *n* (%)	9 (16.4%)	1 (1.8%)	0.008	5 (17.9%)	1 (3.6%)	0.084	4 (14.8%)	0 (0.0%)	0.038
b5401 Carbohydrate metabolism [29]	HbA1 >7%, *n* (%)	23 (41.8%)	14 (25.5%)	0.07	9 (32.1%)	5 (17.9%)	0.217	14 (51.9%)	9 (33.3%)	0.169
b7302 Lipid metabolism [30]	LDL 55–70 mg/dL, *n* (%)	6 (10.9%)	8 (14.5%)	0.571	3 (10.7%)	5 (17.9%)	0.442	3 (11.1%)	3 (11.1%)	1
	LDL 71–115 mg/dL, *n* (%)	20 (36.4%)	4 (7.3%)	<0.001	12 (42.9%)	1 (3.6%)	<0.001	8 (29.6%)	3 (11.1%)	0.091
	LDL > 116 mg/dL, *n* (%)	25 (45.5%)	0 (0.0%)	<0.001	12 (42.9%)	0 (0.0%)	<0.001	13 (48.1%)	0 (0.0%)	<0.001
e1100 Food [31]	Alcohol consumption > 10 g (>1 unit), *n* (%)	1 (1.8%)	1 (1.8%)	1	0 (0.0%)	0 (0.0%)	1	1 (3.8%)	1 (3.8%)	1
e1101 Drugs [17]	NSAIDs, *n* (%)	46 (83.6%)	35 (63.6%)	0.017	21 (75.0%)	19 (67.9%)	0.554	25 (92.6%)	16 (59.3%)	0.004
e1109 Products or substances for personal consumption, other specified [18]	Smoking, *n* (%)	19 (34.5%)	5 (9.1%)	0.001	8 (28.6%)	2 (7.1%)	0.036	11 (40.7%)	3 (11.1%)	0.013

Chi-squared test ALT—Alanine aminotransferase; AST—Aspartate aminotransferase; ALP—Alkaline phosphatase; CVD—Cardiovascular disease; eGFR—Estimated glomerular filtration rate; HbA1c—Glycated hemoglobin 1c; HR—Heart rate; LDL—Low-density lipoprotein cholesterol; NOAC—Non-vitamin K antagonist oral anticoagulants; NSAIDs—Nonsteroidal anti-inflammatory drugs; ULN—Upper limit of normal, VKA—Vitamin K antagonist.

**Table 3:** Profile of CVD risk factors as per categories of the ICF classification.

ICF Category		Complete		Severe		Modrate	Mild	No		
Body Functions	Scoring	Percentage Distribution of Occurrence of Qualifiers								
b152 Emotional functions [26]	Depresion BDI	26–63		20–25			10–12	0–9		
	SG	21.80%		78.20%						
	CG	9.30%		90.70%						
	SG Females	25.00%			75.00%					
	CG Females	14.30%		85.70%						
	SG Males	18.50%		81.50%						
	CG Males	3.80%	96.20%							
b134 Sleep functions [6]	Sleep time (h)	<6 and >9						6 to 9		
	SG	36.40%				63.60%				
	CG	21.80%		78.20%						
	SG Females	32.10%				67.90%				
	CG Females	25.00%			75.00%					
	SG Males	40.70%				59.30%				
	CG Males	18.50%		81.50%						
b4100 Heart rate [7]	HR	>80/min						<80/min		
	SG	46.30%					53.70%			
	CG	21.80%		78.20%						
	SG Females	53.60%					46.40%			
	CG Females	35.70%				64.30%				
	SG Males	38.50%				61.50%				
	CG Males	7.40%		92.60%						
b4101 Heart rhythm [8]	Heart Rhythm	Atrial Fibrillation						Normal sinus rhythm		
	SG	27.30%			72.70%					
	CG	9.10%		90.90%						
	SG Females	21.40%		78.60%						
	CG Females	14.30%		85.70%						
	SG Males	33.30%				66.70%				
	CG Males	3.80%	96.20%							
b4150 Functions of arteries [9]	Stenosis (%)	>70		50–69				<50		
	SG	27.30%			27.30%		45.40%			
	CG	1.80%	98.20%							
	SG Females	25.00%			28.60%		46.40%			
	CG Females	3.60%	96.40%							
	SG Males	29.60%				25.90%	44.50%			
	CG Males	100.00%								
b4200 Increased blood pressure [27]	BP (mmHg)	>180/110		>160/90		>140/90	>130/80	<130/80		
	SG	4.50%	15.00%	25.50%		25.00%	30.50%			
	CG	18.50%		34.20%			47.30%			
	SG Females	4.50%	35.50%				45.50%		4.50%	2.50%
	CG Females	20.50%		39.30%			40.70%			
	SG Males	3.80%	30.50%			25.50%	25.00%			7.80%
	CG Males	30.00%				29.30%	40.70%			
b4302 Metabolite-carrying functions of the blood [12,28]	eGFR (mL/min/1.73 m^2^)	<15		15–29		30–59	60–89	>90		
	SG	5.00%	5.90%	89.10%						
	CG	4.10%	5.00%	91.00%						
	SG Females	5.00%	5.70%	89.30%						
	CG Females	4.30%	10.00%	86.00%						
	SG Males	5.00%	6.10%	88.90%						
	CG Males	3.80%	96.20%							
	Bilirubin (ULN)	>2×						<2×		
	ALT/AST/Alkaline phosphatase (ULN)	>3×						<3×		
	SG	9.10%		90.90%						
	CG	1.80%	98.20%							
	SG Females	14.30%		85.70%						
	CG Females	3.60%	96.40%							
	SG Males	3.80%	96.00%							
	CG Males	100.00%								
b4303 Functions related to the coagulation of blood [13]	VKA	YES						NO		
	SG	20.00%		80.00%						
	CG	16.40%		83.60%						
	SG Females	21.40%		78.60%						
	CG Females	25.00%		75.00%						
	SG Males	18.50%		81.50%						
	CG Males	7.40%		92.60%						
	NOAC	YES						NO		
	SG	16.40%		83.60%						
	CG	1.80%	98.20%							
	SG Females	17.90%		82.10%						
	CG Females	3.60%	96.40%							
	SG Males	14.80%		85.20%						
	CG Males	100.00%								
b5401 Carbohydrate metabolism [29]	HbA1 (%)	>7						<7		
	SG	41.80%				58.20%				
	CG	25.50%			74.50%					
	SG Females	32.10%				67.90%				
	CG Females	17.90%		82.10%						
	SG Males	51.90%					48.10%			
	CG Males	33.10%				66.70%				
b7302 Lipid metabolism [30]	LDL-C (mg/dL)	>116		115–71		70–55		<55		
	SG	45.50%					36.40%		10.90%	7.20%
	CG	7.30%		14.50%	78.20%					
	SG Females	42.90%				42.90%			10.70%	3.50%
	CG Females	3.60%	17.90%		78.50%					
	SG Males	48.10%					29.60%	11.10%	11.20%	
	CG Males	11.10%		11.10%	77.80%					
**Environmental factors**										
e1100 Food [31]	Alcohol consumption (g)	>10						<10		
	SG	1.80%	98.20%							
	CG	1.80%	98.20%							
	SG Females	100.00%								
	CG Females	100.00%								
	SG Males	3.80%	96.20%							
	CG Males	3.80%	96.20%							
e1101 Drugs [17]	NSAIDs	YES						NO		
	SG	83.60%							16.40%	
	CG	63.60%					36.40%			
	SG Females	75.00%					25.00%			
	CG Females	67.90%					32.10%			
	SG Males	92.60%								7.40%
	CG Males	59.30%					40.70%			
e1109 Products or substances for personal consumption, other specified [18]	Smoking	YES						NO		
	SG	34.50%				65.50%				
	CG	9.10%		90.90%						
	SG Females	28.60%				71.40%				
	CG Females	7.10%		92.90%						
	SG Males	40.70%				59.30%				
	CG Males	11.10%		88.90%						

ALT—Alanine aminotransferase; AST—Aspartate aminotransferase; ALP—Alkaline phosphatase; BP—Blood pressure; CVD—Cardiovascular disease; eGFR—Estimated glomerular filtration rate; ICH—intracerebral hemorrhage; ICF—International Classification of Functioning, Disability and Health; IS—ischemic stroke; HbA1c—Glycated hemoglobin 1c; HR—Heart rate; LDL-C—Low-density lipoprotein cholesterol; n—size of the sample; NOAC—Nonvitamin K antagonist oral anticoagulants; NSAIDs—Nonsteroidal anti-inflammatory drugs; ULN—Upper limit of normal; VKA—Vitamin K antagonist.

**Table 4:** Differences in the incidence of the total value of CVD risk factors.

	CG vs. SG			Females			Males		
	SG	CG	*p*	SG	CG	*p*	SG	CG	*p*
Mean ± SD	5.5 ± 1.9	2.8 ± 1.5	<0.001	5.4 ± 1.7	3.2 ± 1.5	<0.001	5.6 ± 2.2	2.4 ± 1.4	<0.001
Median	5	3		5	3		6	2	
Min–Max	2–10	0–6		3–9	1–6		2–10	0–5	

CG—control group; CVD—cardiovascular disease; SD—standard deviation; SG—post-ischemic stroke group.

## Data Availability

The data presented in this study are available on request from the first author. The data are not publicly available due to ethical restrictions.

## References

[1] Roth G.A., Forouzanfar M.H., Moran A.E., Barber R., Nguyen G., Feigini V.L., Naghavi M., Mensah G.A., Murray C.J. (2015). Demographic and epidemiologic drivers of global cardiovascular mortality. N. Engl. J. Med..

[2] Skajaa N., Adelborg K., Horváth-Puhó E., Rothman K.J., Henderson V.W., Thygesen L.C., Sørensen H.T. (2022). Risks of Stroke Recurrence and Mortality After First and Recurrent Strokes in Denmark: A Nationwide Registry Study. Neurology.

[3] Mendis S., Abegunde D., Yusuf S., Ebrahim S., Shaper G., Ghannem H., Shengelia B. (2005). WHO study on Prevention of Recurrences of Myocardial Infarction and StrokE (WHO-PREMISE). Bull. World Health Organ..

[4] Stewart J., Manmathan G., Wilkinson P. (2017). Primary prevention of cardiovascular disease: A review of contemporary guidance and literature. JRSM Cardiovasc Dis..

[5] Yuan H.W., Wang C.X., Zhang N., Bai Y., Shi Y.Z., Zhou Y., Wang Y.L., Zhang T., Zhou J., Yu X. (2012). Poststroke depression and risk of recurrent stroke at 1 year in a Chinese cohort study. PLoS ONE.

[6] Cappuccio F.P., Cooper D., D’Elia L., Strazzullo P., Miller M.A. (2011). Sleep duration predicts cardiovascular outcomes: A systematic review and meta-analysis of prospective studies. Eur. Heart J..

[7] Woodward M., Webster R., Murakami Y., Barzi F., Lam T.H., Fang X., Suh I., Batty G.D., Huxley R., Rodgers A. (2014). The association between resting heart rate, cardiovascular disease and mortality: Evidence from 112,680 men and women in 12 cohorts. Eur. J. Prev. Cardiol..

[8] Lip G.Y., Hunter T.D., Quiroz M.E., Ziegler P.D., Turakhia M.P. (2017). Atrial fibrillation diagnosis timing, ambulatory ECG monitoring utilization and risk of recurrent stroke. Circ. Cardiovasc. Qual. Outcomes.

[9] Orrapin S., Rerkasem K. (2017). Carotid endarterectomy for symptomatic carotid stenosis. Cochrane Database Syst. Rev..

[10] Liu L., Wang Z., Gong L., Zhang Y., Thijs L., Staessen J.A., Wang J. (2009). Blood pressure reduction for the secondary prevention of stroke: A Chinese trial and a systematic review of the literature. Hypertens. Res..

[11] Weiner D.E., Tighiouart H., Stark P.C., Amin M.G., MacLeod B., Griffith J.L., Salem D.N., Levey A.S., Sarnak M.J. (2004). Kidney disease as a risk factor for recurrent cardiovascular disease and mortality. Am. J. Kidney Dis..

[12] Pisters R., Lane D.A., Nieuwlaat R., de Vos C.B., Crijns H.J., Lip G.Y. (2010). A novel userfriendly score (HAS-BLED) to assess 1-year risk of major bleeding in patients with atrial fibrillation: The Euro Heart Survey. Chest.

[13] Gerner S.T., Kuramatsu J.B., Sembill J.A., Sprügel M.I., Hagen M., Knappe R.U., Endres M., Haeusler K.G., Sobesky J., Schurig J. (2019). Characteristics in non-vitamin K antagonist oral anticoagulant-related intracerebral hemorrhage. Stroke.

[14] Wu S., Shi Y., Wang C., Jia Q., Zhang N., Zhao X., Liu G., Wang Y., Liu L., Wang Y. (2013). Glycated hemoglobin independently predicts stroke recurrence within one year after acute first-ever non-cardioembolic strokes onset in A Chinese cohort study. PLoS ONE.

[15] Amarenco P., Bogousslavsky J., Callahan A., Goldstein L.B., Hennerici M., Rudolph A.E., Sillesen H., Simunovic L., Szarek M., Welch K.M. (2006). Stroke Prevention by Aggressive Reduction in Cholesterol Levels (SPARCL) Investigators. High-dose atorvastatin after stroke or transient ischemic attack. N. Engl. J. Med..

[16] Ois A., Gomis M., Rodríguez-Campello A., Cuadrado-Godia E., Jiménez-Conde J., Pont-Sunyer C., Cuccurella G., Roquer J. (2008). Factors associated with a high risk of recurrence in patients with transient ischemic attack or minor stroke. Stroke.

[17] Narum S., Solhaug V., Myhr K., Brørs O., Kringen M.K. (2013). Characterisation of non-warfarin-associated bleeding events reported to the Norwegian spontaneous reporting system. Eur. J. Clin. Pharmacol..

[18] Epstein K.A., Viscoli C.M., Spence J.D., Young L.H., Inzucchi S.E., Gorman M., Gerstenhaber B., Guarino P.D., Dixit A., Furie K.L. (2017). Smoking cessation and outcome after ischemic stroke or TIA. Neurology.

[19] Słowik A., Wnuk M., Brzegowy P., Chrzanowska-Wásko J., Golenia A., Łasocha B., Włoch-Kopéc D., Ferens A., Serednicki W., Jarocki P. (2017). Mechanical thrombectomy in acute stroke—Five years of experience in Poland. Neurol. Neurochir. Pol..

[20] Strazzullo P., D’Elia L., Cairella G., Garbagnati F., Cappuccio F.P., Scalfi L. (2010). Excess body weight and incidence of stroke: Meta-analysis of prospective studies with 2 million participants. Stroke.

[21] Arboix A., Jiménez C., Massons J., Parra O., Besses C. (2016). Hematological disorders: A commonly unrecognized cause of acute stroke. Expert Rev. Hematol..

[22] Adams R.J., Chimowitz M.I., Alpert J.S., Awad I.A., Cerqueria M.D., Fayad P., Taubert K.A., American Heart Association/American Stroke Association, Stroke Council and the Council on Clinical Cardiology of the American Heart Association, American Stroke Association (2003). Coronary risk evaluation in patients with transient ischemic attack and ischemic stroke: A scientific statement for healthcare professionals from the Stroke Council and the Council on Clinical Cardiology of the American Heart Association/American Stroke Association. Stroke.

[23] Maritz R., Aronsky D., Prodinger B. (2017). The International Classification of Functioning, Disability and Health (ICF) in Electronic Health Records. Appl. Clin. Inform..

[24] Chen S., Tao J., Tao Q., Fang Y., Zhou X., Chen H., Chen Z., Huang J., Chen L., Chan C.C. (2016). Rater experience influences reliability and validity of the Brief International Classification of Functioning, Disability, and Health Core Set for Stroke. J. Rehabil. Med..

[25] Lucki M., Chlebuś E., Wareńczak A., Lisiński P. (2021). The ICF Classification System to Assess Risk Factors for CVD in Secondary Prevention after Ischemic Stroke and Intracerebral Hemorrhage. Medicina.

[26] Ceccarini M., Manzoni G.M., Castelnuovo G. (2014). Assessing depression in cardiac patients: What measures should be considered?. Depress Res. Treat..

[27] Cuspidi C., Tadic M., Grassi G., Mancia G. (2018). Treatment of hypertension: The ESH/ESC guidelines recommendations. Pharmacol. Res..

[28] Inker L.A., Astor B.C., Fox C.H., Isakova T., Lash J.P., Peralta C.A., Kurella Tamura M., Feldman H.I. (2014). KDOQI US commentary on the 2012 KDIGO clinical practice guideline for the evaluation and management of CKD. Am. J. Kidney Dis..

[29] Inzucchi S.E., Bergenstal R.M., Buse J.B., Diamant M., Ferrannini E., Nauck M., Peters A.L., Tsapas A., Wender R., Matthews D.R. (2015). Management of hyperglycemia in type 2 diabetes, 2015: A patient-centered approach: Update to a position statement of the American Diabetes Association and the European Association for the Study of Diabetes. Diabetes Care.

[30] Schwartz G.G., Steg P.G., Szarek M., Bhatt D.L., Bittner V.A., Diaz R., Edelberg J.M., Goodman S.G., Hanotin C., Harrington R.A. (2018). ODySSEy OUTCOMES Committees and Investigators. Alirocumab and car-diovascular outcomes after acute coronary syndrome. N. Engl. J. Med..

[31] Wood A.M., Kaptoge S., Butterworth A.S., Willeit P., Warnakula S., Bolton T., Paige E., Paul D.S., Sweeting M., Burgess S. (2018). Risk thresholds for alcohol consumption: Combined analysis of individual-participant data for 599,912 current drinkers in 83 prospective. Lancet.

[32] Mozaffarian D., Benjamin E.J., Go A.S., Arnett D.K., Blaha M.J., Cushman M., Das S.R., de Ferranti S., Després J.P., Fullerton H.J. (2016). American Heart Association Statistics Committee; Stroke Statistics Subcommittee. Heart Disease and Stroke Statistics-2016 Update: A Report from the American Heart Association. Circultion.

[33] Blanco-Rojas L., Arboix A., Canovas D., Grau-Olivares M., Oliva Morera J.C., Parra O. (2013). Cognitive profile in patients with a first-ever lacunar infarct with and without silent lacunes: A comparative study. BMC Neurol..

[34] Zhang J., Wang Y., Wang G.N., Sun H., Sun T., Shi J.Q., Xiao H., Zhang J.S. (2011). Clinical factors in patients with ischemic versushemorrhagic stroke in East China. World J. Emerg. Med..

[35] Wang S.L., Wang C.L., Wang P.L., Xu H., Du J.P., Zhang D.W., Gao Z.Y., Zhang L., Fu C.G., Chen K.J. (2016). Resting heart rate associates with one-year risk of major adverse cardiovascular events in patients with acute coronary syndrome after percutaneous coronary intervention. Exp. Biol. Med.

[36] Lewington S., Clarke R., Qizilbash N., Peto R., Collins R. (2003). Prospective Studies Collaboration. Age-specific relevance of usual blood pressure to vascular mortality: A meta-analysis of individual data for one million adults in 61 prospective studies. Lancet.

